# Diagnosis of B-Cell Non-Hodgkin Lymphomas with Small-/Intermediate-Sized Cells in Cytopathology

**DOI:** 10.1155/2012/164934

**Published:** 2012-05-27

**Authors:** Joerg Schwock, William R. Geddie

**Affiliations:** Division of Anatomical Pathology, Department of Laboratory Medicine and Pathobiology, University of Toronto, Toronto General Hospital, Room E11-219, Toronto, ON, Canada M5G 2C4

## Abstract

Fine needle sampling is a fast, safe, and potentially cost-effective method of obtaining tissue for cytomorphologic assessment aimed at both initial triage and, in some cases, complete diagnosis of patients that present clinically with lymphadenopathy. The cytologic diagnosis of B-cell non-Hodgkin lymphomas composed of small-/intermediate-sized cells, however, has been seen as an area of great difficulty even for experienced observers due to the morphologic overlap between lymphoma and reactive lymphadenopathies as well as between the lymphoma entities themselves. Although ancillary testing has improved diagnostic accuracy, the results from these tests must be interpreted within the morphological and clinical context to avoid misinterpretation. Importantly, the recognition of specific cytologic features is crucial in guiding the appropriate selection of ancillary tests which will either confirm or refute a tentative diagnosis. For these reasons, we here review the cytologic characteristics particular to five common B-cell non-Hodgkin lymphomas which typically cause the most diagnostic confusion based on cytological assessment alone: marginal zone lymphoma, follicular lymphoma, mantle cell lymphoma, chronic lymphocytic leukemia/small lymphocytic lymphoma, and lymphoplasmacytic lymphoma. We summarize the most pertinent cytomorphologic features for each entity as well as for reactive lymphoid hyperplasia, contrast them with each other to facilitate their recognition, and highlight common diagnostic pitfalls.

## 1. Introduction

Over the past 25 years, a large number of articles have been published and significant academic discourse has occurred, and continues to occur, around the usefulness and role of cytopathology in the diagnosis of lymphoma [1–3]. Sophisticated ancillary techniques have changed the landscape considerably and now play a major role in the diagnosis of lymphoproliferative disorders. We decided therefore to revisit the cytologic diagnosis of the five most common B-cell non-Hodgkin lymphomas (NHL) with small-/intermediate-sized cells with a renewed focus on morphology itself. In the following article, we will focus on cytologic samples obtained from lymph nodes by the use of small caliber needles, that is, fine-needle sampling/aspiration. We will not discuss the specifics of other specimen types such as samples of body cavity effusions or cerebrospinal fluid.

The primary and most significant role of fine-needle sampling in undiagnosed lymphadenopathy is to triage the patient appropriately for further management which may include subsequent diagnostic procedures such as excisional or core biopsy. Broadly, fine needle specimens can be categorized into neoplastic and nonneoplastic. Among the nonneoplastic specimens, the possible diagnoses include normal lymphoid tissue, nonspecific reactive change, inflammatory changes indicative of a specific process such as suppurative or granulomatous lymphadenitis, or rarely lymphadenopathies of unknown etiology such as Rosai-Dorfman disease. Within the neoplastic category, the most important distinction to be made is between primary lymphoid and secondary metastatic neoplasms. Subsequent considerations include the type and, if possible, the grade of the neoplasm in order to assign the patient to the most appropriate path for further management. In patients with a prior diagnosis of lymphoma additional questions arise such as the potential presence of changes related to therapy, the assessment for transformation/progression from a low-grade lymphoma to a higher grade, the recurrence of previously treated lymphoma, and the exclusion or inclusion of a synchronous or metachronous second malignancy.

The principle advantage of fine-needle sampling, particularly in superficial palpable lymph nodes, is the ease of specimen acquisition, which provides rapid access to diagnostic material not requiring the extensive tissue processing associated with excisional biopsies. Fine needle sampling causes only minimal patient discomfort and has the potential to spare at least a proportion of patients from a surgical procedure if subsequent lymphadenectomy is deemed unnecessary. When small caliber needles are used (25 gauge or 27 gauge), the risk of subsequent histology being compromised in any way by infarction, hematoma, or other artifact is very small. In selected patients with a single accessible enlarged lymph node and with an established diagnosis of lymphoma, the node can be spared from excision for later clinical assessment of a therapeutic response. In patients with multifocal disease multiple fine-needle samples permit the mapping of sites of involvement and planning of the optimal site for excisional biopsy where required. Also, fine-needle sampling maybe the most practical way to assess patients with deep seated lymphadenopathy or those who are too ill to undergo more invasive procedures [4, 5]. Disadvantages of fine-needle sampling of lymph nodes are mainly associated with the nature of the specimen in which architectural features, although not completely absent, are less obvious than in histologic preparations. There is a known potential for sampling error and bias which may be alleviated to some degree by using multiple needle passes into a single lymph node, ultrasound guidance to ensure sampling of different areas of a node, and sampling of multiple lesions in patients that present with more than a single enlarged lymph node. Concurrent core needle biopsy or fine-needle sampling with on-site evaluation and possible conversion to core biopsy have both been advocated to improve specimen adequacy [5–7]. Interestingly, Gong et al. concluded that there was no clear advantage in the diagnosis and classification of small B-cell NHL by adding core needle biopsy to fine-needle sampling [6].

Opponents may argue that fine-needle sampling provides only limited material for ancillary testing. Lymphoid tissues are, however, predisposed to a rich cellular harvest due to a lack in cellular cohesion. Even in the case of a paucicellular specimen, appropriate sample processing will permit subsequent studies with minimal amounts of material. Finally, the technique of fine-needle sampling itself requires a certain amount of training: failure to render a specific cytologic diagnosis is frequently associated with a lack of recognition that the component steps of sample acquisition and handling are simple, but not trivial.

## 2. Prerequisites and Techniques for Lymph Node Cytology

Difficulties associated with the diagnosis of B-cell NHL with small-/intermediate-sized cells by cytomorphology alone are mostly related to the morphologic overlap between the entities as well as with the changes that occur normally in benign, reactive lymph nodes [8, 9]. Some cases may display partial involvement of a lymph node by a neoplastic process, adding to the diagnostic challenge. Familiarity with the spectrum of “normal” changes in a lymph node, and the morphologic findings associated with them is essential. Lymph node morphology may also vary with respect to the body site as exemplified by an increased number of secondary follicles in cervical lymph nodes, the increased occurrence of plasma cells, monocytes, macrophages and mast cells in deep/mesenteric nodes, and the presence of pigment and/or benign cellular inclusions in lymph nodes from body sites such as the mediastinum or axillary region.

The background of a smear produced from a cytologic specimen of a lymph node usually shows an abundance of Söderstrom bodies, mostly referred to as lymphoglandular bodies (LGB), which measure 2–10 micrometers in size and occur both in benign and malignant lymph node aspirates [10]. These structures may be fragments of fragile lymphoid cell cytoplasm avulsed from cells during sampling. Occasionally though, LGBs are found within the cytoplasm of histiocytes which may indicate their existence *in vivo*. The presence or absence of LGBs is not entirely specific for a lymphoid proliferation since occasional lymph node aspirates may display a paucity of these structures whereas samples from some nonlymphoid tumors can have frequent, easily identifiable cytoplasmic fragments [11]. In general, the presence of LGBs in an aspirate, especially if there are more than 20 per high power field, indicates sampling of a lymphoid organ such as lymph node, spleen, thymus, tonsil, or of an extranodal lymphoid proliferation [12, 13]. The number of LGBs may be conspicuously increased if a lymphoid neoplasm is present.

Fine-needle sampling performed with a needle as thin as 27 gauge leaves components of the lymph node architecture intact and, if conventional smears rather than liquid-based cytology are used, these structures will be present on the slide. The most commonly observed part of the native lymph node architecture present in cytological preparations is the lymphohistiocytic aggregate (LHA) or germinal center fragment. LHAs are characterized by the presence of cells of the mononuclear phagocyte system, especially tingible body macrophages (TBMs), which are frequently observed in benign, reactive, or “follicular” lymphoid hyperplasia. Like the secondary follicles of follicular hyperplasia, LHAs are quite variable in size and may be dominated by activated cells (centroblasts) associated with the germinal center reaction. A follicle center fragment (FCF), on the contrary, is an aggregate of lymphoid cells arranged around a meshwork of follicular dendritic cells (“kissing cells”) the interdigitating cytoplasm of which can be highlighted by immunohistochemical staining for CD21 and/or CD23. The latter type of cell aggregate, by some authors referred to as pseudo-LHA [12], may also occur in benign samples, but is seen in increased frequency in smears prepared from lymph nodes involved by follicular lymphoma (FL), constituting an important diagnostic clue in this particular setting. Notably, FCFs in FL are often small and similar in size imparting an “architectural” monomorphism which can be recognized, often grossly, in smear preparations [14]. FCFs may be mimicked in smears occasionally by other cell aggregates such as the densely packed groups of cells associated with proliferation centers, or pseudofollicles, seen in chronic lymphocytic leukemia/small lymphocytic lymphoma (CLL/SLL). In CLL/SLL, no follicular dendritic cells should be visible, but prolymphocytes and paraimmunoblasts will be present. Also, it is important to note that occasional “true” FCFs can be found in mantle cell lymphoma (MCL) and marginal zone lymphoma (MZL), and that LHAs can be seen whenever benign lymphoid tissue is sampled at the same time as lymphoma. Other, less frequently observed cell aggregates in smear preparations may consist of capillary-size vessels with attached lymphoid tissue or occasional aggregates of adipose tissue infiltrated by lymphoid cells.

**Figure 1:**
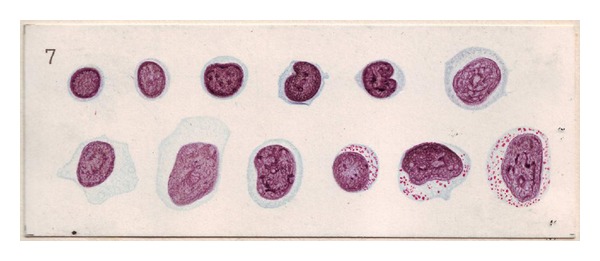
Historical depiction of different lymphoid cell types. Schleip examined the heterogeneity of lymphoid cells in peripheral blood films. This image taken from the atlas published based on his observations illustrates the variable nuclear morphology and cytoplasmic content of benign lymphoid cells in different states of activation.

Readily recognizable normal lymphoid constituents seen in fine-needle samples from benign/reactive lymph nodes include small round lymphocytes (either functionally “naïve” or terminally differentiated memory cells or unstimulated T-cells), centrocytes, centroblasts, immunoblasts, and plasma cells. Common nonlymphoid cell types present in the aspirate are follicular and interdigitating dendritic cells located mainly in germinal centers and paracortex, respectively. Constituents such as histiocytes, macrophages, plasmacytoid monocytes, and epithelioid cells are related to the mononuclear-phagocyte system. Other cell types are mast cells, basophils, neutrophils, eosinophils, endothelial cells, and occasional fat cells. Small lymphocytes have an approximate size of 6–12 micrometers, comparable to a histiocyte nucleus. Practically, erythrocytes and neutrophils are more convenient comparators. The small lymphocyte is usually about twice the size of an erythrocyte and somewhat smaller than the nucleus of a segmented neutrophil. Mature, circulating lymphocytes dominate at the lower end of this size spectrum and activated lymphocytes at the upper end. Large lymphocytes may exceed 20 micrometer size, equivalent to or greater than the diameter of 3 erythrocytes, and larger than a histiocyte or neutrophil nucleus. Lymphocytes that fulfill neither size criterion, usually designated as “intermediate,” include many centrocytes and some centroblasts. It is not unusual for a “small” B-cell lymphoma to be composed mainly of cells that fall into this intermediate size range. This broad spectrum of lymphocyte size and morphology has been long recognized and documented (Figure 1) [15].

The character of the nuclear chromatin, the presence or absence and size of nucleoli, and the character of the cytoplasm (including cytoplasmic granulations) provide better clues to the identification of the position of a lymphoid cell within the functional spectrum than size alone. The typical cytologic features of small round lymphocytes are a regular nuclear contour, dense chromatin, invisible nucleoli, and a narrow rim of cytoplasm. Cells with these properties in medullary cords and sinuses are mainly B cells, but in the paracortex of the lymph node will mainly consist of T-cells. Some small round T-lymphocytes are also present within the germinal centers. The naïve cells of the primary follicle and the mantle zone are very similar but display a slightly more irregular nuclear contour and slightly more open chromatin. B-cells of the marginal zone are characterized by slightly irregular nuclear contours and moderate amounts of pale cytoplasm. Cell types of the lymphoid lineage recognizable in fine-needle specimens include centrocytes, centroblasts, prolymphocytes/(para-)immunoblasts, plasmablasts, plasmacytoid lymphocytes, plasma cells, and monocytoid lymphocytes. Overlap between these cell types occurs, so that it may be difficult to definitively classify some individual cells based on cytomorphology alone. Overall, the small round lymphocyte is always the most frequent cell in reactive lymph nodes. However, the predominance or increased occurrence of any of these cell types, including the small round lymphocyte, should lead at least to the consideration of a neoplastic process as part of the differential diagnosis.

The success of fine-needle cytology is dependent on samples of high cellularity and excellent technical preparation. Several articles and textbooks have detailed descriptions of the technique of fine-needle sampling with aspiration [16]. Briefly, fine-needle sampling of superficial nodes with suction is performed using a 23–27 gauge needle fitted to a 10 mL syringe which may be attached to a one hand grip syringe holder for convenience. Larger needles should be avoided since they will cause considerable admixture of the sample with peripheral blood. Local anesthesia is rarely required for the sampling of superficial, palpable lesions which should be almost painless. Local anesthesia, however, is used in cases where penetration of pleura or peritoneum is necessary. The sampling of a superficial lesion is performed with the mass immobilized between two fingers while one or two needle passes consisting of several (5–10) strokes each are performed. At this stage, the sampling is for the most part due to detachment of cells and tissue fragments by the cutting edge of the needle as described in the method published by Zajdela et al. [17] whereas excessive aspiration may increase the blood content of the sample. The material obtained in this way is often sufficient for several smears and a cell suspension for ancillary studies. Nonpalpable lesions are sampled with the aid of imaging modalities such as ultrasonography or computer tomography.

**Figure 2:**
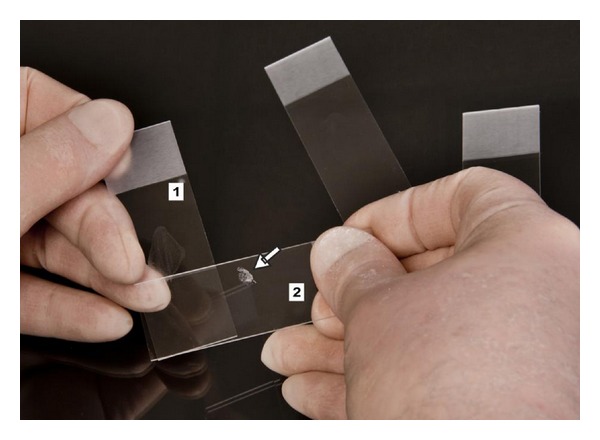
Hand position during the one-step smear with touch-off which allows multiple smears to be produced from a single fine-needle sample. The sample is expelled from the needle and placed near the frosted end of the “receiver” slide (1). The “spreader” slide (2) is then lowered in a hinge-like fashion onto the receiver slide from above, allowing the sample to disperse by capillary action, and then drawn down the length of the receiver slide producing the characteristic “bullet shape with feather edge.” If using the touch-off technique, the spreader slide is lowered to pick up a small amount of the specimen and then turned over so that a clean glass surface can be used to spread the remaining sample on the receiver slide. The picked up sample on the reverse of the spreader slide (arrow) is then used to generate a second smear.

The obtained sample is then placed on a glass slide and a “splitting” or “touch-off technique” can be used to generate several matching smears from the same sample (Figure 2). After the sample is expelled onto the slide, the smear is produced by even and thin spreading of the cells with a second “spreader” slide using the so-called “one-step” technique [18]. During this step, it is important not to apply too much pressure which will destroy fragile cells. Ideally direct smears are subsequently processed using both Papanicolaou and Romanowsky-type stains on paired slides after alcohol fixation and air-drying, respectively. The two staining methods highlight distinct cellular features thereby producing complimentary diagnostic information. In general, alcohol fixation with Papanicolaou stain emphasizes nuclear detail whereas air-drying with Romanowsky-type stains demonstrates the cytoplasm more effectively. Also, Romanowsky-type stains differentially color DNA a metachromatic purple and RNA blue. Diff-Quik, a proprietary “quick” stain, or other nonproprietary modified staining methods are frequently used and are particularly suitable for immediate specimen assessment in situations where confirmation of specimen adequacy is crucial [19]. It should be noted, though, that the higher concentration of methylene blue present in Diff-Quik and similar rapid stains can sometimes cause fine blastic chromatin to appear darker and more mature than it otherwise would, and produce the appearance of “pseudonucleoli” in cells if staining is performed on an incompletely dried smear. May-Grünwald-Giemsa (MGG) stain provides more subtle coloration of nuclei and is superior for staining of cytoplasmic granulations. MGG is highly pH-dependent and may be difficult to standardize. Finally, cell suspensions are produced by aspiration of sterile physiological saline, phosphate-buffered saline solution, or cell culture medium from a tube through the sampling needle into the syringe. The cell suspension can then be used for ancillary testing purposes and either directly submitted for flow or slide-based cytometry or, after further processing as cytospin preparation or cell block, for immunocytochemistry or fluorescence *in-situ* hybridization (FISH). Cell suspensions are ideal material for more specialized molecular methods that may be required for diagnostic and research purposes including polymerase-chain reaction (PCR) and gene expression profiling. Very small amounts of residual cell suspension can be expelled onto an FTA card or paper for indefinite storage of DNA.

## 3. Differential Diagnosis of B-Cell NHLs Composed of Small-/Intermediate-Size Cells

A sequential approach to the examination of fine-needle samples from lymph nodes includes the initial assessment of cellularity and adequacy of the sample. As with samples from other sites, an absolute definition of adequacy is difficult. Pambuccian and Bardales propose a minimum number of 40 lymphoid cells per high power field in the area of highest cellularity as criterion to consider an aspirate as adequate in cases not showing granulomas or metastatic malignancy [12]. By this definition, an extensively hemodiluted specimen would be considered inadequate for the purpose of establishing a complete diagnosis, although it still might yield useful information. Other adequacy criteria related to the quality of the cytologic preparation include a uniform, thin smear resulting in a “bullet shape with feather edge” appearance, lack of involvement of the slide margins, and absence of extensive artifacts [12]. The slide should be examined grossly to determine if the cells smeared diffusely or focally form small or large aggregates. Next, there should be an assessment of the slide background which, in the case of reactive lymph nodes or small B-cell NHLs, should contain numerous LGBs without presence of extraneous elements. Depending on the anatomic site sampled, however, a variety of noncellular material may be present such as anthracotic pigment in mediastinal nodes sampled by endobronchial ultrasound with transbronchial needle aspiration or melanin pigment in axillary lymph nodes involved by dermatopathic lymphadenopathy. The subsequent steps include a low power assessment of the lymphoid cell population for its (a) composition (mono-/dimorphic *versus* polymorphic), (b) presence, density, and nature of cell aggregates (FCF *versus *LHA), and (c) any cell predominance or presence of atypical cells (small *versus* intermediate *versus* large cells). The final step is the assessment of the sample at high power during which the specific nature of the lymphoid population is elicited through recording of the cytologic features of any predominant cell population with or without admixed minor cellular component(s).

In the following section, we will review the cytologic features of B-cell lymphomas characterized by a predominance of cells with small to intermediate size and consider possible diagnostic pitfalls. In the context of the most pertinent clinical scenarios, we will discuss areas of morphologic overlap and important differential diagnoses.

### 3.1. Reactive Lymphoid Hyperplasia

**Figure 3:**
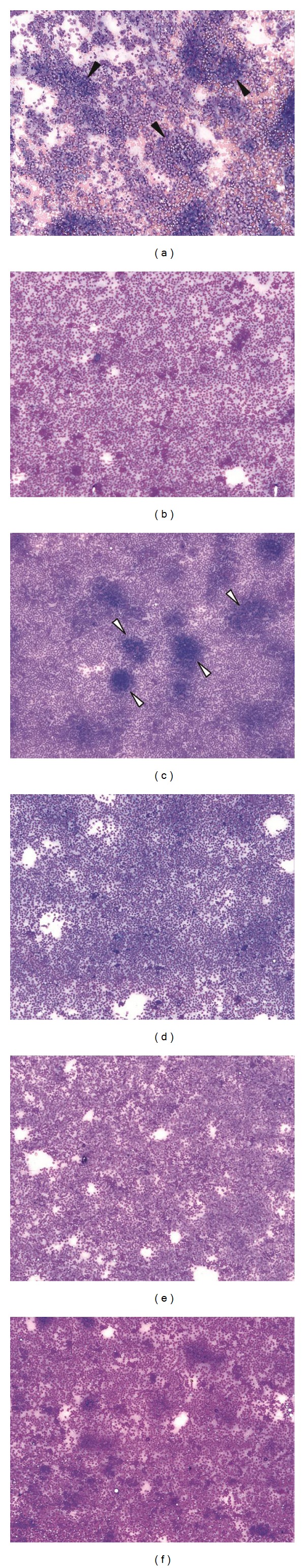
Smears produced from lymph node fine-needle samples and examined at low power. Low power assessment shows the differences in cell composition and the presence or absence of cell aggregates in reactive lymphoid hyperplasia (a), marginal zone lymphoma (b), follicular lymphoma (c), mantle cell lymphoma (d), chronic lymphocytic leukemia/small lymphocytic lymphoma (e), and lymphoplasmacytic lymphoma (f). Note the variable size and frequency of aggregates in reactive lymphoid hyperplasia (black arrowheads). Compare with the monomorphism of the small aggregates in follicular lymphoma (white arrowheads) and the absence or vague delineation of aggregates in the other lymphoma cases. Also, there is a markedly more polymorphic cell composition in reactive lymphoid hyperplasia as compared to the other cases. (d: Diff-Quik, all other: May-Grünwald-Giemsa; 10x).

**Figure 4:**
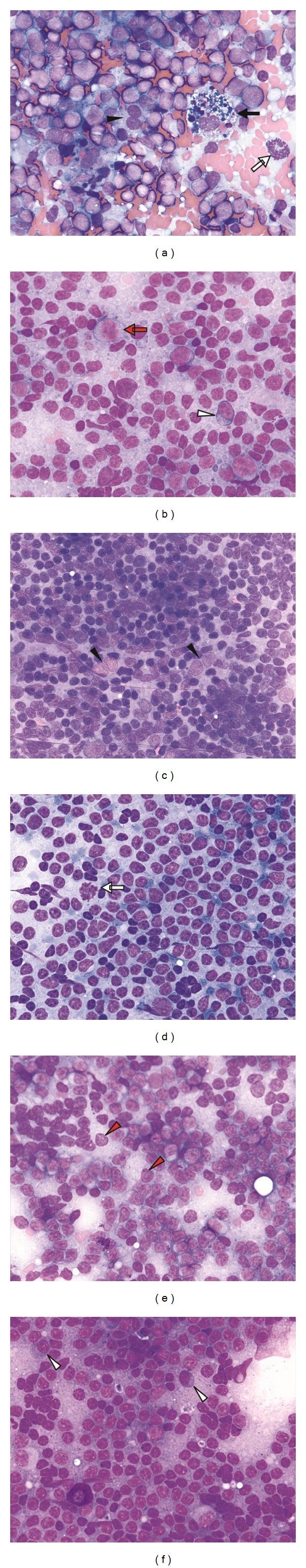
Smears produced from lymph node fine-needle samples and examined at high power. High power assessment shows the differences in cell composition between reactive lymphoid hyperplasia (a) and the cytological features of the neoplastic cells in marginal zone lymphoma (b), follicular lymphoma (c), mantle cell lymphoma (d), chronic lymphocytic leukemia/small lymphocytic lymphoma (e), and lymphoplasmacytic lymphoma (f). Follicular dendritic cells (black arrowheads), tingible body macrophages (black arrow) and mitoses (white arrows) are present within a lymphohistiocytic aggregate seen in reactive lymphoid hyperplasia. Monocytoid B-cells (red arrow) and plasmacytoid cells (white arrowheads) are often found in marginal zone lymphoma. Follicular dendritic cells are a prominent feature of follicle center fragments present in samples of follicular lymphoma. These follicle center fragments do not show the other constituents seen in the lymphohistiocytic aggregates of a reactive lymph node. Mitoses are also a frequent finding in mantle cell lymphoma, but are not commonly seen in the other types of B-cell non-Hodgkin lymphoma illustrated here. Prolymphocytes (red arrowheads) are often found in areas of vague nodularity present in smears of chronic lymphocytic leukemia/small lymphocytic lymphoma and identified by a nucleolus which has a tinctorial quality similar to the cytoplasm of the cell. Plasmacytoid cells and mast cells (f: left lower image quadrant) are constituents seen in lymphoplasmacytic lymphoma. (d: Diff-Quik, all other: May-Grünwald-Giemsa; 63x).

Reactive lymphoid hyperplasia (RLH) is the most common cause for a polymorphic lymphoid cell pattern in a fine-needle sample and is mostly associated with benign, reactive and reversible lymphadenopathy. The histologic correlate is an expansion of the sinusoidal, follicular, paracortical, or medullary area of a lymph node leading to corresponding changes in cellular composition. Two distinct patterns of RLH exist with (a) follicular hyperplasia as correlate of a B-cell response and (b) paracortical hyperplasia as correlate of a T-cell response [12]. Both patterns are characterized by a spectrum of differentiation of the lymphoid cells with numerous small mature lymphocytes in the background resulting in a polymorphic impression at low power assessment (Figure 3(a)). In follicular hyperplasia, LHAs of variable size with conspicuous TBMs are often evident at low power, and a mixture of centrocytes, centroblasts, and plasma cells is prominent (Figure 4(a)). Immunoblasts are dominant in paracortical hyperplasia, particularly viral lymphadenopathy. It is important to note, however, that the two reaction patterns, follicular and paracortical hyperplasia, often present together. Also, temporal changes in the cell composition occur until the RLH resolves and are associated with concomitant cytologic variations.

In most cases the etiology of the RLH remains unknown. RLH most frequently affects children and young adults. Bacteria, viruses, chemicals, and drugs are the most common inciting agents [12]. Considering patient age and cytomorphology, the differential diagnosis in most cases will include Hodgkin lymphoma, T-cell lymphoma, and FL. Depending on the degree of cytomorphologic change, ancillary testing may need to be employed to exclude those mimics. After cytologic diagnosis of RLH an attempt should be made to establish the cause by appropriate history, serology, or other investigations, but as mentioned above the etiology may remain undetermined. If resolution of the lymphadenopathy does not occur, excisional biopsy is warranted.

### 3.2. Marginal Zone B-Cell Lymphoma

Nodal MZL is a rare neoplasm (1.5–1.8% of all lymphoid neoplasms [20]) which, similar to RLH, presents with a distinctly polymorphic cytologic pattern [21, 22]. By definition, nodal MZL presents without evidence of extranodal or splenic disease. However, the similarly rare splenic MZL and the more common extranodal MZL of mucosa-associated lymphoid tissue (MALT) may resemble nodal MZL in cytomorphology. LHAs may be present, and only careful examination at higher power may suggest the presence of monotonous neoplastic cells within a background resembling nonspecific RLH. This may be particularly problematic in partially involved nodes. In contrast, in cases with follicular colonization, the smear may contain numerous FCFs. Rare cases show a monotonous intermediate-size cell pattern at low power assessment. The neoplastic cells may be exclusively monocytoid, but more typically are cytologically polymorphic with monocytoid cells occurring alongside small lymphocyte- and centrocyte-like cells [12] as well as with elements showing plasma cell differentiation [20] (Figures 3(b) and 4(b)). The cytomorphology of such samples, therefore, leads frequently to a broad differential diagnosis including RLH as well as the spectrum of lymphomas with small and intermediate-sized cells [23]. Finally, an important potential pitfall is peripheral T-cell lymphoma which can mimic MZL morphologically [24].

MZL usually affects the middle-aged and elderly, although it may occur in children. Clinical history is important with regard to multiple sites of involvement including intraabdominal lymphadenopathy and presence of constitutional symptoms. Consideration should be given to the possibility of nodal dissemination of extranodal MZL which occurs in approximately one third of the cases [20]. The clinical course is indolent, but transformation to a large B-cell lymphoma may occur. Ancillary studies are particularly important in making the diagnosis of MZL, but cases in which the reactive cells obscure the neoplastic B-cell population even by immunophenotyping may occur [25]. A further complication is the well-documented occurrence of small clonal, but nonneoplastic, B-cell populations in some of the chronic inflammatory conditions, such as Sjögren's syndrome, which may be associated with extranodal marginal zone (MALT) lymphoma.

### 3.3. Follicular Lymphoma

FL grade 1-2 is characterized by a monotonous to somewhat dimorphic pattern, respectively. FL grade 3A or 3B is characterized by a dominant population of large cells resembling centroblasts and is, therefore, not discussed here. The dimorphic pattern may initially cause the false impression of a polymorphic cell composition at low power. Regardless of grade, smears often give the low-power impression of a nodular pattern which can be an important clue to the diagnosis (Figure 3(c)). At higher power, the nodules correspond to numerous FCFs populated by small to intermediate-sized centrocytic cells with cleaved nuclei and clumped chromatin (Figure 4(c)). Cells with deeply cleaved, apparently bilobed nuclei can be conspicuous [26]. Larger centroblasts characterized by noncleaved nuclei, less dense chromatin, and a narrow rim of dense cytoplasm are rare in grade 1 FL, but occur with increasing frequency in grade 2 where both cell populations may be present in equal numbers. Rarely, LHAs with TBMs may occur which should not distract from the overall impression. LHAs and mitotic activity are both uncommon, and the presence of either should raise suspicion for a reactive process which then needs to be distinguished from partial lymph node involvement by lymphoma. Of note, a rare signet-ring variant of FL exists and is due to immunoglobulin stored within the cytoplasm of the neoplastic cells. A diagnostic challenge can be posed by diffuse FL which is rare and characterized by a lack of follicular architecture in which case FCFs would be absent. The differential diagnosis in this situation would include insufficient sampling of a FL displaying a focal diffuse component.

FL accounts for approximately 20% of all lymphomas, and affects adults and elderly individuals. FL occurring before the age of 20 years is rare. Characteristically there is often widespread nodal involvement at diagnosis. Histologic grading of FL is based on the number of centroblasts per high-power field (HPF, 0.159 mm^2^) counted in ten HPFs within different representative follicles [20]. Similar grading can be performed on cytological preparations but is not universally accepted [27, 28]. The vast majority of FLs are grade 1 or 2. As distinction between these two is poorly reproducible and not clinically important, the diagnostic terminology usually reported is “follicular lymphoma, grade 1-2/3.” Although an indolent lymphoma, transformation to diffuse large B-cell lymphoma (DLBCL) is more frequent than with other low-grade lymphomas [12] and areas of DLBCL are present in a substantial proportion of grade 3 FLs. Fine-needle sampling plays an important role in the initial assessment of FL since it allows sampling of multiple sites of involvement so that the optimal site for excisional biopsy (if required) can be determined. Similarly, in a case of suspected transformation multiple fine-needle samples improve the chances of finding and documenting progression.

### 3.4. Mantle Cell Lymphoma

MCL is characterized, in contrast to the lymphomas considered previously, by a distinctly monomorphic cell composition [29, 30]. Histologically three patterns of MCL occur: diffuse, vaguely nodular, and mantle zone pattern, the latter with preservation of preexisting follicles. Accordingly, smears may be diffuse, show poorly formed FCFs, or contain intact LHAs. The mantle zone pattern, however, is rarely seen in cytologic material. Most commonly, smears consist of a highly monotonous population of lymphoid cells interrupted only by small round lymphocytes and occasional histiocytes [12] (Figure 3(d)). At higher magnification, the cells have subtly irregular nuclear contours reminiscent of centrocytes, dispersed to clumped chromatin, mostly inconspicuous nucleoli, and moderate amounts of cytoplasm. Mitotic figures may be observed, and this feature can be used together with monomorphism and nuclear contour irregularities in differentiating MCL from other lymphomas [31] (Figure 4(d)). Diagnostic challenges may be posed by any of the variants of MCL. Marginal zone-like MCL and small cell MCL may be confused with MZL or small lymphocytic lymphoma, respectively. The blastoid and pleomorphic variant both display a more aggressive clinical behavior and may enter the differential diagnosis in cases sampled at relapse which can show increased nuclear size, pleomorphism and mitotic activity [20]. The latter two variants, again, can be confused with DLBCL.

MCL comprises 3–10% of lymphomas and predominantly occurs in middle-aged to older males. Lymphadenopathy, hepatosplenomegaly, bone marrow, and peripheral blood involvement are frequent. The median survival is 3–5 years and the majority of patients cannot be cured [20]. Grading of MCL is not performed. However, the estimation of the proliferative fraction either by counting mitotic figures or Ki67 staining has shown prognostic impact [32]. Given the different prognosis of MCL, ancillary testing to discriminate the above-mentioned variants of MCL from their less aggressive counterparts is crucial.

### 3.5. Small Lymphocytic Lymphoma/Chronic Lymphocytic Leukemia

**Figure 5:**
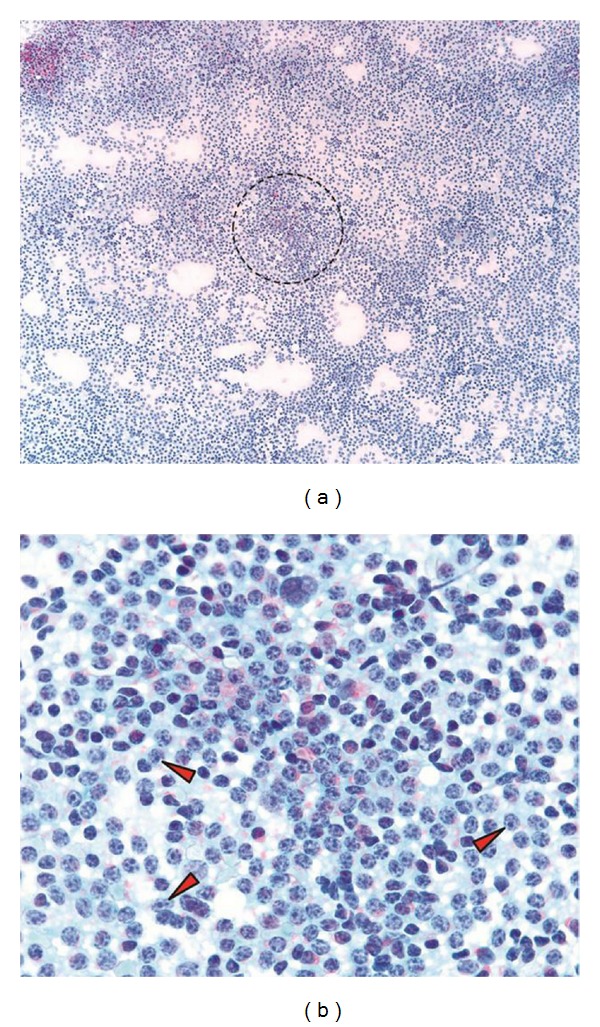
Fine needle samples of chronic lymphocytic leukemia/small lymphocytic lymphoma examined after Papanicolaou stain. Several ill-defined cellular aggregates (circle) are visible at low power inspection giving the impression of vague nodularity (a). High-power examination reveals the peculiar chromatin distribution and presence of numerous prolymphocytes (red arrowheads) within this area of increased cell density (b). Note the coarse distribution of the chromatin and tinctorial quality of the nucleoli in the prolymphocytes in this stain. ((a) and (b): Papanicolaou; (a): 10x, (b): 63x).

SLL/CLL is characterized by a monomorphic cellular aspirate often without conspicuous nodularity or presence of aggregates. Thicker areas of a smear occasionally show poorly defined aggregates of cells that may correspond to the proliferation centers associated with SLL/CLL (Figures 3(e) and 5(a)). In Romanowsky-stained smears these are appreciated as darker zones at low power, whereas in H&E-stained histologic sections proliferation centers appear as pale zones. Proliferation centers differ from FCFs in that they do not contain follicular dendritic cells. At higher magnification, small lymphoid cells with smooth-contoured nuclei and minimal cytoplasm predominate (Figure 4(e)). The chromatin frequently has a peculiar coarsely clumped appearance best appreciated on Papanicolaou-stained slides which led to the original designation as “cellules grumelées” [33, 34] (Figure 5(b)), a term which refers to clotted milk or cheese curd. “Soccer balls” might be a better modern appellation. Most cases have interspersed intermediate-sized prolymphocytes with dispersed chromatin and obvious nucleolus as well as paraimmunoblasts with broader, pale cytoplasm, vesicular chromatin, and large central nucleolus (Figures 4(e) and 5(b)). Both cell types are most often seen in those ill-defined aggregates corresponding to the proliferation centers, and their presence provides a clue towards the diagnosis. A common artifact in smears from patients with CLL/SLL is the presence of smudge cells, also referred to as basket cells or shadow cells of Gumprecht, which are lymphoid cells stripped of their cytoplasm with the nuclear material dispersed on the slide. This artifact, however, is not specific to CLL/SLL. In samples with more than a few cells with plasmacytoid features, lymphoplasmacytic lymphoma (LPL) needs to be considered in the differential diagnosis to CLL/SLL. Another important differential diagnosis is the benign, minimal enlarged lymph node with a high proportion of small lymphocytes and few secondary follicles which, at first glance, may cause a similarly monotonous impression as CLL/SLL [35].

CLL/SLL is, in general, an indolent disease and occurs at a mean age of 65 years. It is the most common lymphoproliferative disorder of adults in Western countries and shows a slight male preponderance. The disease is rarely seen in individuals of Asian descent. Fine-needle sampling is often done to exclude Richter's transformation to DLBCL which occurs in 2–8%. Transformation to Hodgkin lymphoma occurs in less than 1% of the patients. Immunoglobulin gene mutation status and specific cytogenetic abnormalities correlate with disease prognosis [20], and can be examined using cytologic material.

### 3.6. Lymphoplasmacytic Lymphoma

Fine-needle specimens from patients with LPL show a monomorphic, rarely more polymorphic cell composition without a significant number of cell aggregates. The hint to the diagnosis is an admixture of small lymphocytes and plasmacytoid cells (Figures 3(f) and 4(f)). Mature plasma cells may be present, but if dominant, plasmacytoma should be considered in the differential diagnosis. Intranuclear Dutcher bodies and intracytoplasmic Russell bodies may be seen in the neoplastic cells, and occasional multinucleated cells can be present. Mast cells are often seen and are diagnostically helpful when frequent, but they may also occur in other B-cell lymphomas with small-/intermediate-sized cells [22]. In contrast to CLL/SLL, prolymphocytes and paraimmunoblasts are absent which is useful in excluding this differential diagnosis. Importantly, LPL may be indistinguishable from MZL both histologically or cytologically in which case ancillary testing and correlation with clinical presentation are indispensable.

LPL is a rare lymphoma and most frequently occurs in elderly patients with slight male predominance. The disease is often associated with a paraprotein of IgM type, but this is not required for diagnosis. Bone marrow involvement and IgM monoclonal gammopathy are the diagnostic features of Waldenström macroglobulinemia found in a significant subset of patients with LPL. Autoimmune phenomena, hyperviscosity, cryoglobulinemia, coagulopathies, and/or neuropathy characterize the clinical course in a significant proportion of these patients. The lymphoma is typically indolent, but transformation to DLBCL and, rarely, Hodgkin lymphoma occurs in a small number of cases [20].

## 4. The Role of Ancillary Testing in Small B-Cell Non-Hodgkin Lymphoma

Due to the morphologic overlap between different B-cell NHLs with small-/intermediate-sized cells as well as with RLH, ancillary testing plays a central role in their workup [34, 36]. This is true regardless of the method with which the specimen was obtained: excisional biopsy or fine-needle sample. Although smears can be used for immunocytochemistry, it is much more practical to use cell suspensions which can be submitted directly for flow- or slide-based cytometry, or for further processing as cytospins or cell blocks [37]. Each of these methods has advantages and disadvantages, and their use is guided by the particular clinical circumstance and, importantly, by the cytomorphology of the sample which is the basis for a tentative diagnosis. Because material for ancillary testing may be limited, the initial morphological assessment becomes a crucial step and determines if a particular investigative pathway will ultimately lead to the correct diagnosis. Flow cytometry has become indispensable in the diagnosis and classification of B-cell NHLs with small-/intermediate-sized cells [38, 39]. Some institutions have developed expertise in slide-based cytometry which can be used with specimens of very low cellularity and yields immunophenotyping results similar to flow cytometry [14, 40, 41]. However, flow cytometry protocols using ten or more colors can be similarly informative with extremely paucicellular specimens.

The selection of the antibodies, again, is guided by cytomorphology, clinical history, and the amount of material available for testing. A stepwise approach may be employed which includes, at a minimum, kappa, lambda, CD19, CD20, CD3, CD5, CD10, and CD23 for the initial assessment. Additional markers such as CD4, CD8, CD7, CD2, CD43, and FMC7 may be included upfront or subsequently. A kappa : lambda ratio exceeding a threshold between 4 : 1 to 6 : 1 or a lambda : kappa ratio exceeding 3 : 1 on flow cytometry are generally sufficient to prove light chain restriction, that is, monoclonality [12, 42]. The exact thresholds depend on the cut-off(s) set by the individual laboratory. However, in most cases of NHL, these ratios will be clearly surpassed. Flow- or slide-based cytometry are ideal for markers with cell surface expression and invaluable for the assessment of antigen coexpression. Also, some intracellular antigens such as Bcl-2 can be assessed by flow cytometry after cell permeabilization. In contrast, the nuclear expression of Cyclin D1 is best examined using standardized immunohistochemical staining protocols on cell block sections. MIB-1 as an indicator of the proliferative activity may aid in the classification of a B-cell lymphoma according to its expected biological behavior and can be evaluated by immunocytochemistry [43].

Since normal lymph nodes show a predominance of T-cells, a large proportion of CD20-positive B-cells in a fine-needle sample will raise the suspicion of lymphoma. Expression of CD5 and CD23 is indicative of CLL/SLL, the presence of Bcl-2 in a population of germinal center cells positive for CD10 or Bcl-6 is seen in FL, and staining for Cyclin D1 is diagnostic for MCL. LPL will usually lack CD5 and CD23 and show expression of CD38 as well as presence of cytoplasmic immunoglobulin which distinguishes this entity from CLL/SLL. Immunophenotyping by flow cytometry may be the most informative test in cases of MZL where it can reveal a population of B-cells with light chain restriction which, by cytomorphology, may be obscured by a reactive background [38]. Variations may occur with any of the mentioned immunophenotypic markers as exemplified by a range of CD23 expression in CLL/SLL [44]. Consequently, a high level of vigilance is required as well as interpretation of the flow cytometric data in the context of morphological and clinical information. Limitations to the use of flow cytometry, which occur particularly with lymphoid neoplasms that have a rich background of benign cells such as in Hodgkin lymphoma or that have particularly fragile cells such as in some large cell lymphomas, are not usually encountered in B-cell NHLs with small-/intermediate-sized cells.

Cytogenetic testing, most commonly FISH, can be used to demonstrate genetic abnormalities which are either (a) recurrent and diagnostic for a specific lymphoma type, (b) occur at an increased frequency, thus supporting a tentative diagnosis, or (c) confer prognostic information [45]. Trisomy of chromosome 12 is most commonly seen in CLL/SLL where it occurs in approximately 25% of the cases [12]. Trisomy 12 and to a greater extent deletions on chromosomes 11, 17, and 6 are associated with a poorer prognosis in CLL/SLL whereas an isolated deletion of 13q14.3 is a positive prognostic marker. MCL is characterized in almost all cases (>95%) by translocation *t*(11;14)(q13;q32) which involves the genes *IGH* and *CCND1*. Although rare cases may have an alternative translocation, the diagnosis of MCL should be made with caution in cases that are *t*(11;14) negative [46]. Slightly less frequent is the translocation *t*(14;18)(q32;q21) which is seen in approximately 80% of FLs and results in rearrangement of *IGH* and *BCL2*. In contrast, translocation *t*(11;18)(q21;q21) which affects the *API2* and *MALT1* genes is only present in MZL of the extranodal sites (MALT), but not in nodal MZL. Trisomies of chromosome 3 and 18 are nonspecific, but may support the diagnosis of MZL in the appropriate context. There are marked variations in the rate with which these genetic alterations can be detected depending on the anatomic site involved by lymphoma with reported frequencies for *t*(11;18), trisomy 3, and trisomy18 ranging between 0–56%, 11–75%, and 0–25%, respectively [20]. No specific cytogenetic abnormalities have so far been found for LPL leaving cytomorphology and immunophenotyping as primary diagnostic tools. Molecular studies, mostly PCR-based assays for clonality and translocation detection with or without sequencing, are, at the present time, rarely used in the diagnostic workup of B-cell NHLs with small-/intermediate-sized cells. Their application will depend on specific situations where an attempt to classify a neoplasm with the aforementioned techniques has left questions unanswered.

## 5. Summary

The current World Health Organization (WHO) classification of tumors of the hematopoietic and lymphoid tissues emphasizes cellular morphology in addition to immunophenotypic and genotypic characteristics [20]. Since a fine-needle sample, if properly obtained and processed, provides excellent material for both cytomorphologic assessment and ancillary testing, the acceptance of cytology as diagnostic tool in lymphoma diagnosis has increased considerably. Much progress has been made since the publication by Chhieng et al. [9] who, like various other groups, emphasized the difficulty inherent in the cytologic recognition of B-cell NHLs, especially those with predominant small cell component. They reported that these low-grade lymphomas accounted for a significant percentage of false-negative or indeterminate cases. However, they also stated that the diagnosis of malignant lymphoma could be made in 82% of their cases with a significant contribution occurring through the use of immunophenotyping which led to a substantial improvement in diagnostic accuracy. Remarkably, when they compared the results of the histologic assessment based on REAL with their cytologic diagnosis, there was agreement in 85% of the cases. In a similar vein, Wakely Jr [8] reported a sensitivity of >80% and specificity of >90% in the majority of studies focused on the cytologic diagnosis of NHL. However, these results may reflect the best possible scenario which may not be accomplished in each setting [3].

Although much progress has been made over the past decade and despite the development of sophisticated methods for ancillary testing, there is still significant skepticism towards the role of cytology in this setting, and excisional biopsy is frequently regarded as essential for an accurate initial diagnosis and classification of malignant lymphoma [47]. Some of this skepticism is attributed to the overlap in morphologic features between B-cell NHLs with small-/intermediate-sized cells as well as with benign reactive and inflammatory lymphadenopathies. In fact, the significant impediments to fine-needle sampling as stand-alone approach to NHL diagnosis were discussed in a recent commentary which also stressed the need for interdisciplinary collaboration, multiparametric testing and on-site specimen adequacy assessment as requirements for optimal results [3]. Undoubtedly there are situations where even a tentative diagnosis can only be rendered with great difficulty. Such cases, however, are not unique to cytological specimens, and this morphologic ambiguity is part of the reason why ancillary testing, in particular immunophenotyping studies, are considered mandatory in the diagnosis of lymphoproliferative disorders. It is important to emphasize, though, that ancillary studies are crucially guided by the initial cytologic evaluation of the sample which also provides the framework for the proper interpretation of the immunophenotypic and molecular testing results.

Some of the cytologic features of the B-cell NHLs reviewed here have been recognized for some time, whereas others are less well known or have been applied somewhat more variably depending on the training of the individual cytopathologist. An example is the recognition of FCFs in cases of FL initially reported by Suh et al. [48], and their distinction from LHAs based on the presence of follicular dendritic cells alone in the former versus TBMs, follicular dendritic cells and activated cells in the latter. A stepwise approach as summarized in Table 1, which includes a low power assessment for cellularity, monomorphism, and predominant cell size with an astute examination for residual architectural features, that is, cell aggregates, followed by a detailed interrogation of the cytologic features at high power will yield the largest amount of information on which a tentative diagnosis can be built, and has the least potential to cause diagnostic confusion.

Awareness of the diagnostic limitations and pitfalls is of key importance in advancing the acceptance for a cytologic diagnosis of lymphomas. Other sampling techniques including excisional biopsy should be considered where the cytologic diagnosis is equivocal or lymphadenopathy persists without definitive cause. Situations in which other sampling modalities may be given preference include, but are not limited to, suspected T-cell lymphoma, Hodgkin lymphoma and composite lymphomas [49]. Excisional biopsies may also be necessary to satisfy criteria for participation in a clinical trial or for purposes of tissue banking for potential future diagnostic and therapeutic developments. Finally, histologic examination may also be necessary due to persistent controversies such as those related to the grading of FL. On the other hand, excisional biopsy in SLL/CLL is probably never warranted, unless Richter's transformation is suspected, and it can be argued that in the case of the indolent, but currently incurable, B-cell NHL with small-/intermediate-sized cells under clinical observation alone, excisional biopsy is best left for a point in the illness when therapeutic intervention becomes necessary.

## 6. Conclusion

With the advent of sophisticated techniques for ancillary testing and with the emphasis on cytomorphologic and immunophenotypic characteristics for the classification of NHLs, there has been a resurgence in interest in cytologic diagnosis of lymphoproliferative disorders. The cytomorphologic features of B-cell NHLs with small-/intermediate-sized cells in many cases are sufficiently characteristic to permit at least some degree of differentiation. The assessment of these features can be applied in a stepwise or algorithmic fashion to discriminate between entities and to arrive at a final differential diagnosis which can be resolved with additional testing. Clinical data, immunophenotyping and cytogenetics provide the context for cytomorphology, and permit accurate diagnoses to be rendered even in this challenging area. Hence, we conclude that the medical community has come closer to realizing the full potential of fine-needle cytopathology.

## Figures and Tables

**Table 1:** Summary of the cytologic features of B-cell non-Hodgkin lymphomas with small-/intermediate-sized cells.

	Gross	Low Power			High Power							
	Smear	Composition	Aggregates	Cell Size	Nuclei	Nucleoli	Chromatin	Cytoplasm	Mitotic activity	Plasma/-cytoid Cells	Mast Cells	Other Cells

RLH	Large and small aggregates	Polymorphic	LHA and FCF are key features	Small to intermediate	N/A	N/A	N/A	N/A	Present	Present	Present	Complete spectrum
MZL	Large and small aggregates	Polymorphic	LHA and FCF possible	Intermediate	Smooth to mildly irregular	Inconspicuous to large	Open to clumped	Conspi-cuous, abundant in monocytoid forms	Low	Present	Present	Large activated cells, histiocytes, immunoblasts
FL Grade 1/2	Numerous small aggregates	Monomorphic to dimorphic (grades 1-2)	Repetitive FCF common	Intermediate	Irregular, bilobed or “divided” nuclei common	Inconspicuous	Open to clumped	Scant	Low	Absent	Present	Centroblasts
MCL	Vague aggregates	Markedly monomorphic	Vague aggregates (FCF/LHA possible in mantle zone pattern)	Intermediate (small and large cell variants occur)	Subtly irregular	Inconspicuous to large	Dispersed to clumped	Variable, moderate	Possible	Absent/ Rare	Infrequent	Scattered EH present, PLC and PIB *absent *
SLL/CLL	Vague aggregates	Monomorphic	Vague aggregates (proliferation centers)	Small	Smooth	Absent/In-conspicuous	Coarse -clumped	Scant	Low	Infrequent	Infrequent	PLC, PIB, smudge cells, rarely RS cells
LPL	Vague aggregates	Mono-, rarely polymorphic	Rare/ Absent aggregates	Small	Smooth, possible Dutcher bodies	Absent/In-conspicuous	Coarse–clumped, “cart-wheel”	Scant to plasma-cytoid, Russell bodies	Low	Common	Present, often frequent	PLC and PIB *absent *

CB: centroblasts, EH: epithelioid histiocytes, FCF: follicle center fragments, FDC: follicular dendritic cell, FL: follicular lymphoma, LHA: lymphohistiocytic aggregate, LPL: lymphoplasmacytic lymphoma, MCL: mantle cell lymphoma, MZL: marginal zone lymphoma, PLC: prolymphocytes, PIB: paraimmunoblasts, RLH: reactive lymphoid hyperplasia, RS: Reed-Sternberg, SLL/CLL: small lymphocytic lymphoma/chronic lymphocytic leukemia; N/A: not applicable.
